# Computer-generated structured electronic medical records are preferable to conventional medical records for patients with acute abdominal pain - a prospective, double-blinded study

**DOI:** 10.1007/s10916-022-01852-w

**Published:** 2022-08-26

**Authors:** Leena Saaristo, Mika T. Ukkonen, Erkki-Ville Wirta, Sannamari Kotaluoto, Matleena Lammi, Johanna M. Laukkarinen, Satu-Liisa K. Pauniaho

**Affiliations:** 1 Department of Surgery, Seinäjoki Central Hospital, Seinäjoki, Finland; 2 Faculty of Medicine and Health Technology, Tampere University, Tampere, Finland; 3 Department of Gastroenterology and Alimentary Tract Surgery, Tampere University Hospital, Tampere, Finland; 4 Emergency Division, Tampere University Hospital, Tampere, Finland; 5 Department of Adolescent Psychiatry, Tampere University Hospital, Tampere, Finland

**Keywords:** Medical records, Medical Records Systems, Computerized, Abdominal pain, Quality of Health Care

## Abstract

**Objectives:**

Structured medical records improve readability and ensure the inclusion of information necessary for correct diagnosis and treatment. This is the first study to assess the quality of computer-generated structured medical records by comparing them to conventional medical records on patients with acute abdominal pain.

**Materials and methods:**

A prospective double-blinded study was conducted in a tertiary referral center emergency department between January 2018 and June 2018. Patients were examined by emergency department physicians and by experience and inexperienced researcher. The researchers used a new electronical medical records system, which gathered data during the examination and the system generate structured medical records containing natural language. Conventional medical records dictated by physician and computer-generated medical records were compared by a group of independent clinicians.

**Results:**

Ninety-nine patients were included. The overall quality of the computer-generated medical records was better than the quality of conventional human-generated medical records – the structure was similar or better in 99% of cases and the readability was similar or better in 86% of cases, p < 0.001. The quality of medical history, current illness, and findings of physical examinations were likewise better with the computer-generated recording. The results were similar when patients were examined by experienced or inexperienced researcher using the computer-generated recording.

**Discussion:**

The quality of computer-generated structured medical records was superior to that of conventional medical records. The quality remained similar regardless of the researcher’s level of experience. The system allows automatic risk scoring and easy access for quality control of patient care. We therefore consider that it would be useful in wider practice.

## Introduction

Acute abdominal pain is one of the most common reasons for emergency department (ED) visits, and can be a diagnostic challenge even for the most experienced physicians [1]. While radiological studies are often required, a good clinical evaluation including history-taking and thorough physical examination often leads to a diagnosis [2–5]. It was already observed in a study conducted in 1976 that using structured forms for collecting data on patients with abdominal pain improved diagnostic accuracy and quality of care [6].

Patient records traditionally include the information that the attending physician chooses to include. Certain headlines are generally used to create structure in the records, but the content under these headings varies depending on the physician’s style. The text may lack information and be difficult to read when the structure of the text is not standardized.

Providing the patient with optimal treatment requires good quality of medical records. Computer-generated structured medical records guide physicians to record data in such way that it is easy to read and contains all the information needed to make the diagnosis and treat the patient. It can also include risk assessment algorithms and other tools for the physician to use in diagnostics. An earlier study has shown that using double-choice or multiple-choice questions guarantees maximal capture of information [7].

Computer-generated structured medical records have not been widely studied for treating patients with abdominal pain and so far, there is no convincing evidence on the benefits of such methods [8]. Therefore, this study assessed the quality of computer-generated structured medical records by comparing them to conventional human-generated medical records.

## Materials and Methods

A prospective double-blinded study was conducted in Tampere University Hospital ED between January 2018 and June 2018. Patients were eligible if they visited the ED due to acute abdominal pain. Only adult patients (≥ 18 years) were included.

## Structured Electronic Medical Records System

A new electronical medical records was developed for ED use only. A private information technology company (Cinia Oy, Finland) was responsible for the design and programming of the new system. Surgeons from the study group were responsible for the contents of the software; i.e. the questions included in the template and the text the program produces on the basis of physicians’ choices. It was designed for ease of use in the ED setting and also to work on mobile devices such as tablet computers. The new program was integrated with the existing electronic medical record system used in Tampere University Hospital.

## Study Design

All patients were routinely examined by ED physicians (either surgeons or acute care physicians) working in a tertiary care university hospital. In our normal practice these physicians dictate medical records. Dictations are transcribed into text by medical transcriptionists. The physicians performing the initial examinations were unaware of the ongoing research. ED staff was informed that there is another older research going, in which patients are examined by researchers and urine samples are collected. After the initial examination, the patients received information sheets and consent forms. Patients who agreed to participate in the study were then examined by the researchers who were unaware of dictated medical records and earlier findings. There were two researchers. The patients were examined by either an inexperienced preclinical phase medical student or by an experienced resident surgeon. Prior to the study the medical student was only taught how to perform a clinical examination on a patient with abdominal pain. The researchers examined and interviewed the patients using the program developed for the study. To ensure study objectivity the researchers were also unaware of the findings and medical records of the initial examination.

## Data

After the examination, the system automatically generated a structured medical record, while the conventional records were dictated by the physician. The new system used discrete variables, such as age, gender, comorbidities, and symptoms prior to the emergency visit to generate structured medical records in natural language. Some of the information was mandatory, e.g. location of the pain, presence of hernias etc. The researcher was able to correct the text if needed before it was saved in the database.

The medical records were divided into three separate sections. These included medical history, history of current illness and findings of the physical examination. The computer-generated medical records were stored in an external database.

### Data Analysis

Both conventional and computer-generated medical records were anonymized and printed, including the diagnoses. Records of laboratory and radiological examinations were removed. An expert group of three consultant surgeons who did not participate design of this system and were not examining patients in the ED reviewed the medical records individually. A Likert scale (1–5; 1: very poor, 2: poor, 3: fair, 4: good, 5: excellent) was used for the assessment. The assessment included eight questions:

- Overall assessment of the quality (1) and structure (2) of the medical records.
- Assessment of how medical history (3), present illness (4) and physical examinations (5) were documented in the medical records.
- Assessment of whether medical history (6), present illness (7) or physical examinations (8) contained unnecessary information.

The reviewers were unaware of whether the medical records were computer-generated or conventional.

### Statistical Analysis

All statistical analyses were performed using SPSS Statistics version 22 for Windows (IBM Corp, Armonk, NY, USA). Mean assessments of each reviewer and differences between conventional and computer-generated medical records were calculated. We used the Pearson χ^2^ test to test categorical variables and the Kruskal-Wallis test for continuous variables. Interrater reliability was analyzed by using Fleiss Kappa (three reviewers, Likert scale). Kappa values < 0.40 indicated as poor agreement, 0.40–0.74 as moderate and 0.75-1.00 good agreement [9]. Statistical significance was set at p < 0.05.

## Ethical Aspects

The study was conducted according to the Helsinki Declaration and institutional review board approval was obtained (R18539).

## Results

Ninety-nine patients (median age 56 [range 21–93] years, males 43%) were included in the study. Typical diagnoses included non-specific abdominal pain (31%), appendicitis (16%), diverticulitis (14%) and cholecystitis (13%), as presented in Table 1.

**Table 1:** Comparison between computer-generated structured medical records and conventional human-generated medical records for patients with acute abdominal pain (n = 99). Tampere University hospital, January 1st – June 30th 2018

Diagnosis	%	Computer-generated structured records similar or better than conventional				
		Medical history	Present illness	Physical examination	Structure	Readability
Non-specific abdominal pain	31%	77% (24/31)	65% (20/31)	90% (28/31)	100% (31/31)	84% (26/31)
Appendicitis	16%	81% (13/16)	100% (16/16)	94% (15/16)	100% (16/16)	100% (16/16)
Diverticulitis	14%	79% (11/14)	64% (9/14)	86% (12/14)	100% (14/14)	86% (12/14)
Cholecystitis	13%	46% (6/13)	92% (12/13)	92% (12/13)	92% (12/13)	85% (11/13)
Urological disorder	6%	83% (5/6)	83% (5/6)	68% (4/6)	100% (6/6)	67% (4/6)
Pancreatitis	4%	50% (2/4)	25% (1/4)	100% (4/4)	100% (4/4)	100% (4/4)
Hernias	2%	50% (1/2)	100% (2/2)	100% (2/2)	100% (2/2)	100% (2/2)
Miscellaneous	13%	62% (8/13)	77% (10/13)	92% (12/13)	100% (13/13)	77% (10/13)
**All patients**	**100%**	**71% (70/99)**	**76% (75/99)**	**90% (89/99)**	**99% (98/99)**	**86% (85/99)**

**Fig. 1:**
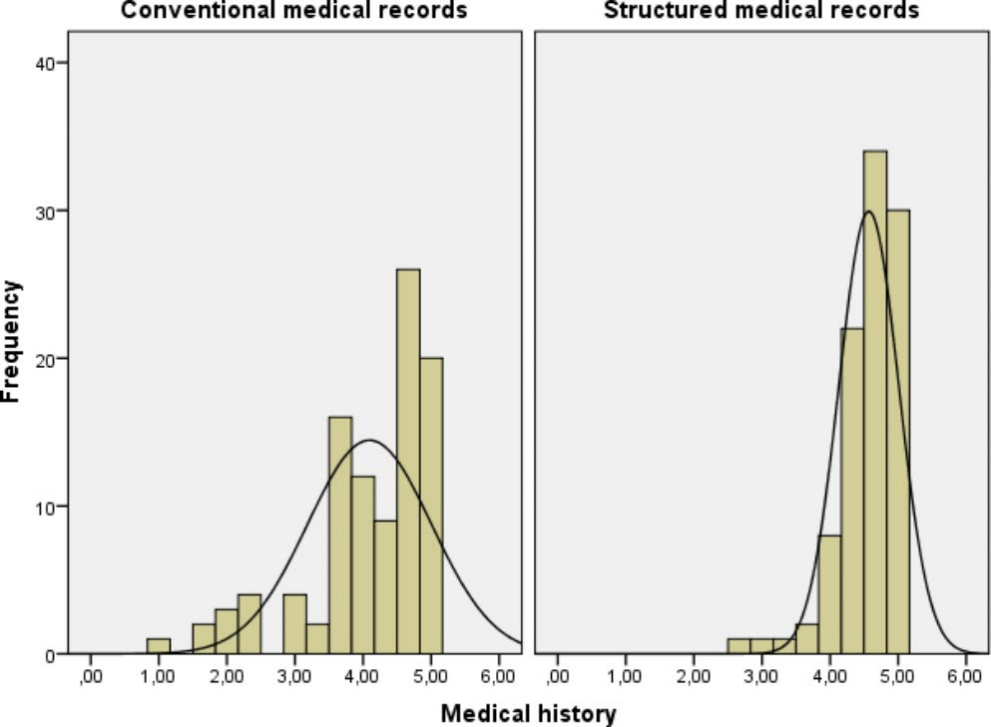
Reviewers’ assessments of patient records (medical history) produced by the old system and the new computer assisted system on a scale from one to five (1 being very poor and 5 being excellent)

**Fig. 2:**
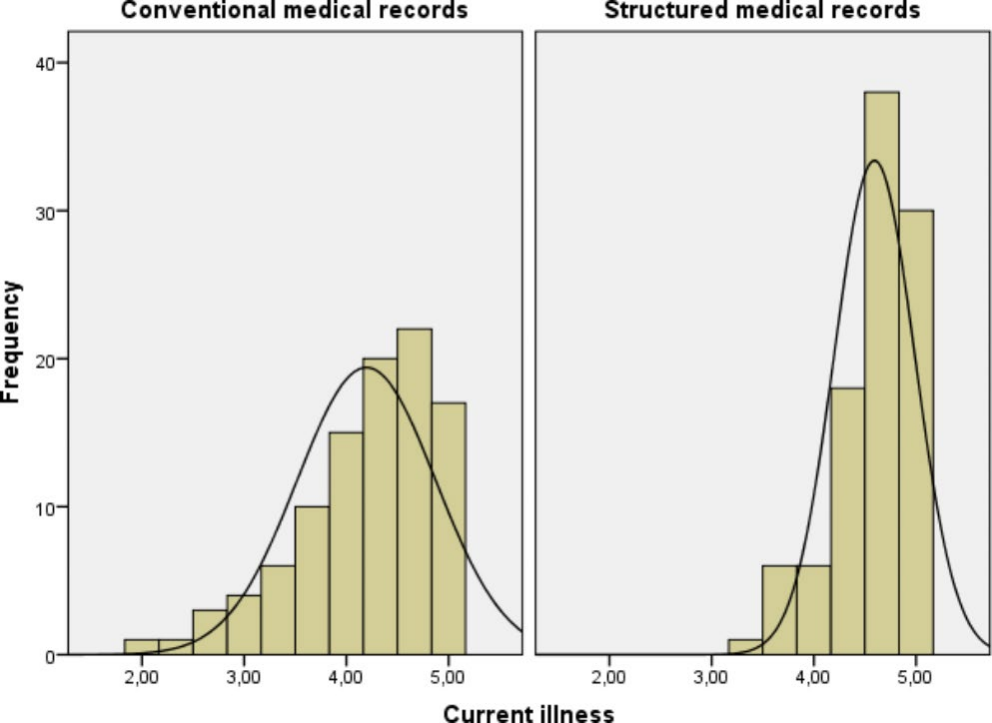
Reviewers’ assessments of patient records (current illness) produced by the old system and the new computer assisted system on a scale from one to five (1 being very poor and 5 being excellent)

**Fig. 3:**
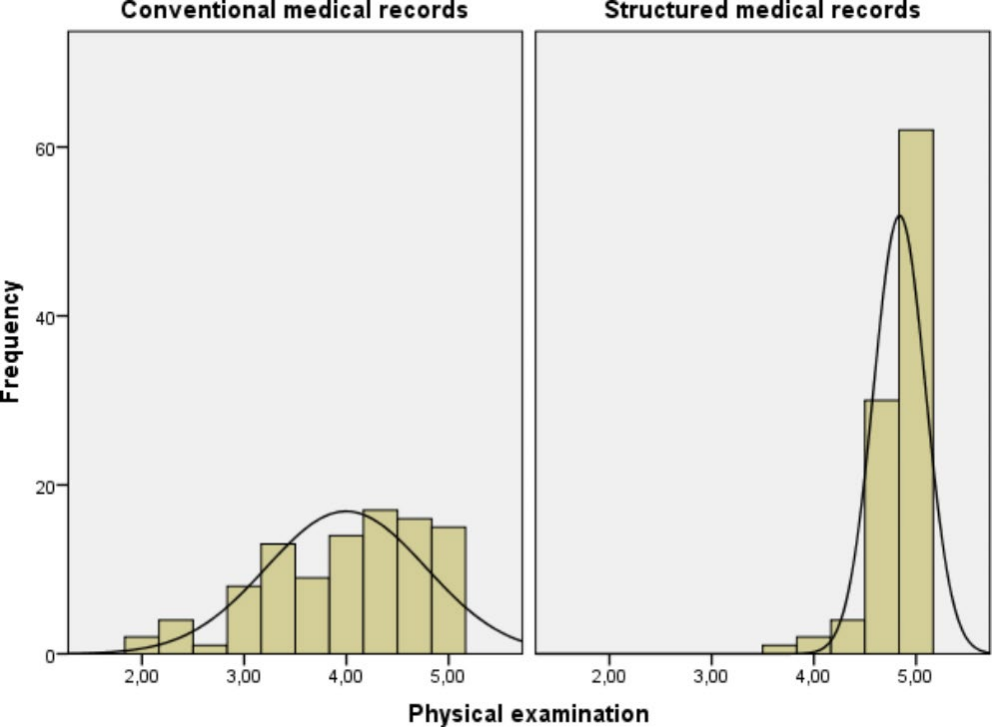
Reviewers’ assessments of patient records (physical examination) produced by the old system and the new computer assisted system on a scale from one to five (1 being very poor and 5 being excellent)

**Fig. 4:**
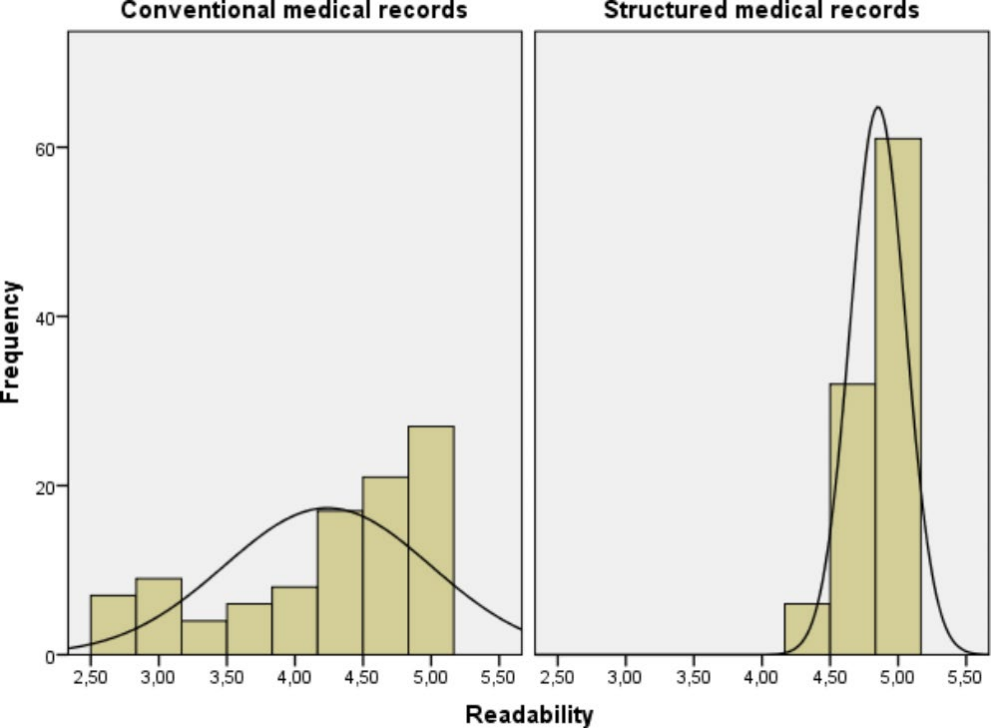
Reviewers’ assessments of patient records (readability) produced by the old system and the new computer assisted system on a scale from one to five (1 being very poor and 5 being excellent)

**Fig. 5:**
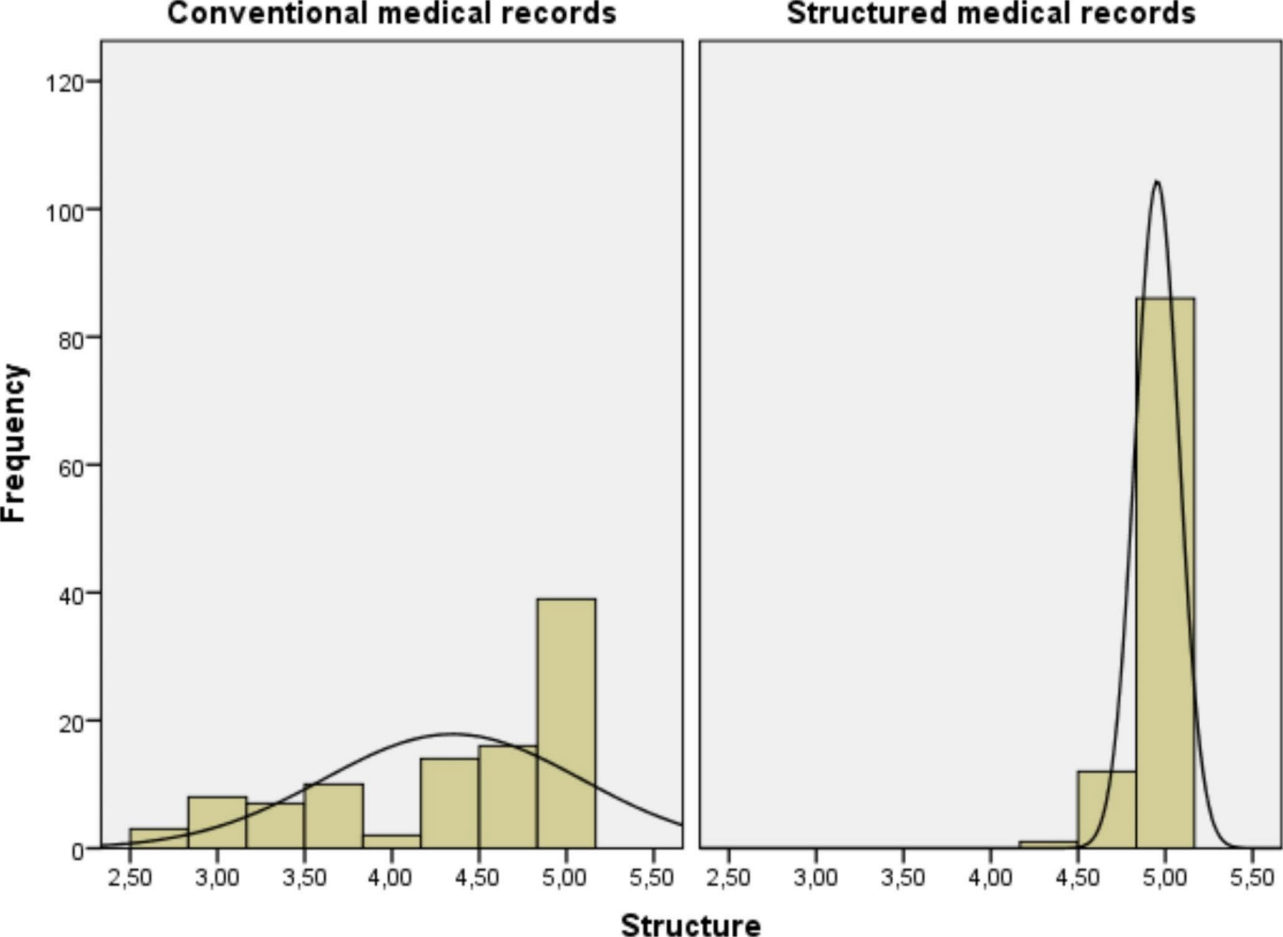
Reviewers’ assessments of patient records (structure) produced by the old system and the new computer assisted system on a scale from one to five (1 being very poor and 5 being excellent)

Reviewers’ assessments are illustrated in Figs. 1, 2, 3, 4 and 5. Overall, level of agreement among raters was good, with Fleiss’ kappa coefficient interpretations ranging from “moderate” to “good”. The raters assessed that the quality of the computer-generated records was better than the quality of the human-generated medical records – the structure of the medical records was similar or better in 99% of cases (worse in only one case) and overall readability was similar or better in 86% of cases (worse in 14 cases). The quality of documenting medical history, current illness, and the findings of physical examinations was better overall with the new system, as shown in Table 3. However, the new system provided slightly inferior medical records for patients with acute cholecystitis.

The reviewers reported that the computer-generated medical records contained unnecessary information less often than the conventional medical records. With the new system medical history, current illness, and physical examinations contained unnecessary information in 13%, 3% and 10% cases, whereas the respective shares with conventional medical records were 30%, 48% and 31%.

The assessments were similar when the records by inexperienced and experienced researchers were compared, as shown in Table 2. The overall structure, readability, as well as the quality of recorded medical history, current illness, and findings of the physical examinations was better in computer-generated medical records than in the conventional medical records even when the patients were examined by an inexperienced researcher.

**Table 2:** Computer generated-medical records similar or better than conventional medical records. Medical records generated electronically by a medical student and a resident surgeon compared to conventional medical records made in the ED. (n = 99)

	Computer-generated medical records similar or better	
	**Medical student**	**Resident surgeon**
Medical history	72% (42/58)	68% (28/41)
Present illness	72% (42/58)	81% (33/41)
Physical examination	98% (57/58)	93% (38/41)
Structure	98% (57/58)	100% (41/41)
Readability	78% (45/58)	98% (40/41)

**Table 3:** Assessment (scale from 1 to 5, 1 being very poor and 5 being excellent) of the conventional and computer generated electronic medical records system (n = 99)

	New system	Old system	New better, %	p-value
Medical history	4.57 (± 0.44)	4.09 (± 0.91)	71% (70/99)	< 0.001
Current illness	4.59 (± 0.39)	4.20 (± 0.68)	76% (75/99)	0.055
Physical examination	4.84 (± 0.25)	4.00 (± 0.78)	90% (89/99)	0.047
Structure	4.95 (± 0.13)	4.35 (± 0.74)	99% (98/99)	0.102
Readability	4.85 (± 0.20)	4.24 (± 0.76)	86% (85/99)	0.132

## Discussion

Poor quality of information in medical records may be associated with poor quality of care and may thus be associated with higher rates of adverse events. In this study we show that the computer-generated structured medical records were of better quality, contained less unnecessary information and were more reader-friendly than the conventional medical records.

In case of abdominal emergencies, the specific symptoms may be vague, and in some case even absent. While radiological studies are often required, a good clinical evaluation including history-taking and thorough physical examination often leads to a diagnosis. We hypnotize that better quality of texts produced by the new system reduces treatment delays and thus reduces morbidity. The authors believe that information system, such as the one studied, can guide clinicians to choose the appropriate laboratory and radiological tests. There are currently ample risk assessment scores and tools available. However, using these in clinical practice can be quite challenging as we have to search for the scores or calculators from various sources (such as internet sites). The same problem arises when integrating systematic preoperative risk assessment. So far, only simple scores, such as the ASA grade (American Society of Anesthesiologists physical status classification system) [10], are used systematically worldwide. Using a computerized system such as ours, we can integrate more complex scoring systems, in addition to scores for specific acute conditions (e.g. adult appendicitis score) [11] into clinical practice as the program calculates the risk scores automatically. Furthermore, structured electronic medical records enable us to gather continuously so-called “big data”, which can be used in future to incorporate multiple variables into new risk assessment models. While it can be easily improved, during the study the system calculated only the National Early Warning Score (NEWS) [12], and gave recommendations, for example, if a patient required immediate attention or ICU care.

We used clinician-driven design process for our system. In this design process invited end users participated at various levels, including the clinical design, testing and pilot user. Hence, we consider the system better meets the needs not only of clinicians’ in the ED setting but also later on hospital wards and in outpatient clinics. We found it encouraging that the clinicians experience did not affect the quality of the medical records. The quality of records produced by the medical student using the new system was better than that of the conventional records produced by experienced physicians working in the ED. We consider that additional applications, such as use in primary health care or in education, can be easily found. As the system gathers data on patients’ physical and functional ability, it enables follow-up of patients’ functional ability and the impact of acute illnesses on this. As data on patients’ condition and symptoms is gathered systematically, the progress of the disease and onset of new symptoms can be easily monitored on subsequent ED visits.

Even though the design and implementation of the system cause financial costs it achieves indirect savings by reducing morbidity and delays in treatment, as well as direct savings by reducing the need for clerical personnel in the hospital to transcribe the physicians’ dictations. Furthermore, it allows us easy access for purposes of quality assurance and a perfect platform to gather research data. At the beginning of this project one of the main targets was to make clinicians’ work easier. Our system can be implemented on tablet computers and therefore can be easily used while examining the patient. It can also generate the referral to radiological imaging which can be accomplished rapidly if needed. We therefore consider the system to improve the patient flow in the ED. As our system was developed in close co-operation with clinicians and the design process was user-centered, the final product was easy and fast to use.

There were some limitations to this study. The system was tested only on patients with acute abdominal pain. However, the diagnostics of acute abdominal pain is considered to be challenging, and thus it is likely that the system will work well on other patient groups with less complex complaints. Lower quality of dictated medical records could be related to different documentation styles of ED physicians and work-related factors, such as stress. In this study all the physicians working in emergency department were experienced. Therefore we consider our results illustrate advantages of the system. The results might have been even better if inexperienced physicians had examined patients. While there are some obvious strengths in the system, some possible limitations might exist. Diagnosis such as acute cholecystitis are typically more straightforward than other less common conditions. Therefore systems might be more suitable for some diagnosis. More data will be required. Even though we consider the system to be suitable for all ED patient groups, more research on other patient groups is required. Also, the number of patients on whom the system was tested was small.

## Conclusion

The quality of the structured medical records was better than that of the conventional medical records. Our system is easy and quick to use and may reduce costs of care by optimizing the transformation of the patient data and thus improving patient safety. It may also improve the patient flow in the ED. The system also enables systemic use of risk assessment tools.
